# Comparing clinical protocols for the treatment of human rabies: the Milwaukee protocol and the Brazilian protocol (Recife)

**DOI:** 10.1590/0037-8682-0352-2020

**Published:** 2020-11-06

**Authors:** Leandro Augusto Ledesma, Elba Regina Sampaio Lemos, Marco Aurélio Horta

**Affiliations:** 1 Fundação Oswaldo Cruz, Programa de Pós-Graduaçao Stricto Sensu em Medicina Tropical, Rio de Janeiro, RJ, Brasil.; 2 Fundação Oswaldo Cruz, Instituto Oswaldo Cruz, Laboratório de Hantaviroses e Rickettsioses, Rio de Janeiro, RJ, Brasil.; 3 Fundação Oswaldo Cruz, Instituto Oswaldo Cruz, Plataforma NB3, Rio de Janeiro, RJ, Brasil.

**Keywords:** Rabies, Milwaukee Protocol, Recife Protocol

## Abstract

**INTRODUCTION:**

Rabies is a major and seriously neglected public health problem worldwide. A treatment consisting of supportive therapy with the use of drugs that show antiviral activity is called the Milwaukee Protocol. In Brazil, this protocol was adapted to the national reality and called the Recife Protocol. In this study, we compared the Milwaukee Protocol with the Recife Protocol, assessing the differences and how these differences may change the course of clinical management.

**METHODS:**

We searched electronic databases for the use of anti-rabies treatments. A total of 65 articles were published between 2004 and 2019.

**RESULTS:**

The protocols have similarities in care related to rabies patients and are important for the treatment of patients in intensive care units. Both protocols indicate deep sedation, antiviral use, constant concern with electrolyte balance, and vasoconstriction related to the condition. Many differences were observed in this study. For the Milwaukee Protocol, sedation should be gradually removed after the eighth day, and on the twelfth day, the patient should be without sedation. In the Recife Protocol, in order to avoid immunomodulation, it is recommended to remove sedation according to the titers of neutralizing antibodies to the rabies virus in the cerebral spinal fluid.

**CONCLUSIONS:**

In addition to the differences and similarities raised, our findings indicate that these protocols require a large center for rabies treatment, but the disease most often occurs in places where resources and hospital infrastructure are scarce.

## INTRODUCTION

Rabies is a disease caused by viruses of the genus *Lyssavirus*, a member of the *Rhabdoviridae* family, which primarily infects mammals^1^. The rabies virus (RABV) is enveloped and bullet-shaped, measuring 180 × 75 nm^2^. Although rabies is a vaccine-preventable disease, it accounts for 60,000 deaths a year worldwide. Approximately 15 million people receive some post-exposure prophylaxis (PEP) for rabies annually^3^ ^-^ ^6^. Even with a global distribution of the disease, the largest number of cases occur in Asia and Africa, accounting for 95% of cases, and the main form of contagion in these continents is dog bite^7^. Considering lost years of life, rabies ranks seventh among infectious diseases^8^.

The RABV incubation period is quite variable, and the first symptoms may occur from 20 to 90 days after exposure^9^. Early symptoms are due to viral replication in the dorsal ganglion root and include pain, paresthesia, and/or pruritus. After a short prodromal phase (about 48-72 hours), the patient begins an acute neurological phase, characterized by clinical neurological manifestations. Two clinical forms are recognized in rabies presentation: fury (also called tropical encephalitis) and paralysis^10^. In the furious presentation, the typical symptoms are agitation, hypersalivation, and hydrophobia. The paralytic form causes muscle weakness and paralysis. Both forms tend to progress to coma and death^11^. Symptoms such as hydrophobia and aerophobia have a strong relationship with rabies, but for definitive diagnosis, specific laboratory tests that can differentiate rabies from other kinds of viral encephalitis are required^7^. For rabies diagnosis, patient saliva samples and a hair follicle biopsy can be obtained.

Although rabies has a high mortality rate, there are reports in the literature of patients who survived the disease. Approximately 20 cases are described as rabies survivors worldwide^12^. Two clinical protocols can be used to treat patients with rabies. These protocols are known as the Milwaukee Protocol and the Recife Protocol^13^. In 2003, researchers proposed a therapy for rabies treatment that included vaccination, rabies immunoglobulin, ribavirin, interferon gamma, and ketamine^14^. The following year, a 15-year-old girl with a bat bite on her right hand received a combination of drugs that was believed to cure rabies. This patient had neutralizing antibody titers within 48 h of treatment^11^. She was kept in an induced coma with deep sedation, received amantadine and ribavirin (antivirals) and survived with a few sequelae. This treatment became known as the Milwaukee Protocol. After this first case, the Milwaukee Protocol was used in at least 39 other, similarly treated, cases. The effectiveness of the Milwaukee Protocol and the lethality of rabies cannot be quantitatively estimated due to difficulties in obtaining information about the cases in which it was used. The United States recorded the case of a rabies survivor in which several precepts of the Milwaukee Protocol were applied. This case occurred in an 8-year-old patient from California in 2011^15^. Since its first publication in 2004, the Milwaukee Protocol has undergone some modifications; the latest publication of this protocol is version 6, published in November 2018.

In Brazil, the first case of a patient who survived rabies occurred in Recife in 2008^16^. The patient, a 16-year-old girl, received a treatment similar to that recommended by the Milwaukee Protocol, named the Recife Protocol^13^. This case was similar to the Milwaukee's case, in which the patient suffered a bat bite and received medical attention three weeks after the incident. In 2018, another patient survived rabies in Brazil after undergoing the Recife Protocol. A 17-year-old boy acquired rabies after a bat bite. The aim of this study was to compare the two protocols presented here as well as to analyze the cases in which the protocols were used to evaluate outcome, reports of medications used, incubation time, and hospitalization time of each case, published in the literature.

## METHODS

Comparative analyses were performed between the Milwaukee Protocol, version 6.0, and the Recife Protocol that was administered in human rabies cases during the period of 2004-2019. A comprehensive search was conducted with the PubMed and Google Scholar databases using the following terms: “rabies survivor” and “rabies AND Milwaukee protocol”, and “rabies AND Recife protocol”. First, we identified 65 articles using these terms. After reading the abstract, 26 articles were excluded because they did not fit the aim of this paper, and two other articles were excluded because of duplication. Thus, a total of 37 articles describing 39 cases of human rabies submitted to the Milwaukee Protocol or to Recife Protocol were identified and included in this work.

The information comparing the two protocols was tabulated using a Microsoft Excel spreadsheet, separated into two columns for the Milwaukee and Recife Protocols. The variables collected after reading the protocols were classified according to whether they were related to intensive care of the patient, recommended or not recommended medications, complementary exams that should be requested, and specific exams for rabies. The periodicity exams that should be requested were also tabulated in the data. When one of the protocols does not mention something that is cited in the other, this information has been left blank in the table. D1 was defined as the first day of the protocol, with subsequent days following numerical order. For glycemic control, the recommendation is insulin dripping or regular insulin use as needed, varying from protocol to protocol. For temperature control, the Celsius unit was used.

All information gathered from case reports in which the Milwaukee Protocol was applied were sent to spreadsheets, detailing: the patient’s gender and age; the aggressor animal; dates of transmission; clinical presentation and protocol opening; outcomes; region of occurrence; use of ketamine, amantadine, and/or ribavirin, and sedation; and phylogenetic analysis of the virus related to the case. We excluded cases in which the transmission of the disease occurred after transplantation of organs from a patient with rabies.

## RESULTS

We observed differences between the protocols. Table 1 presents the main aspects of each protocol. We found 39 published cases in which the principles of the Milwaukee Protocol were applied (Table 1). Both protocols draw attention to the importance of electrolyte control in patients. The Recife Protocol draws attention to complications from hypernatremia and hyponatremia. Hypernatremia may be caused by dehydration or diabetes insipidus. In these cases, it is important to define the cause for the correct treatment; an important exam to define the etiology is urinary density. Facing a diagnosis of diabetes insipidus, desmopressin or vasopressin is indicated.

**TABLE 1:** Comparison of Milwaukee and Recife clinical protocols in the treatment of human rabies, from 2004 to 2019.

Clinical feature	Milwaukee	Recife
Vaccine or immunoglobulin	Not recommended	Not recommended
Isolation	Recommended	Recommended
Sedative for aggressiveness	Recommended	Ramsey IV
Sedation withdrawal	Initiate progressive withdrawal after D8*-complete on D12	After 3-5 IU/mL** antibody titers in CSF
Hyponatremia	Attention to D5-D8: Cerebral salt wasting (CSW)	To evaluate diagnoses of SCPS*** and syndrome of inappropriate antidiuretic hormone secretion
Hypernatremia	No recommendations	Treating causes: Dehydration or diabetes insipidus
Glycemic control	Insulin dripping use	Insulin as needed
Diuresis	0.5 mL/(kg∙h)	0.5 mL/(kg∙h)
Temperature	35-37 °C	35-37 °C
Medications		
Ketamine	Recommended	Recommended
Midazolam	Recommended	Recommended
Fentanyl	Contraindicated	Not Recommended
Haloperidol to improve sedation	Recommended	-
Amantadine	Recommended	Recommended
Vitamin C	Indicated 250 mg-500 g IV/day	indicated 1 g IV/day
Sapropterin or Biopterin	Sapropterin 5mg/(kg∙day)	Biopterin 8 mg/kg IV 8/8h
Fludrocortisone or Hydrocortisone	Recommended	Recommended
Complementary exams		
Serum Na+ dosage	Twice a day	Twice a day
Serum Mg++ dosage	Daily between D5 and D8 and D12 and D15	Daily
Serum zinc	Weekly	Weekly
Nuclear magnetic resonance	Recommended	Recommended
Transcranial Doppler	Daily	Daily
Eyes ultrasound	Daily	No comments
Virology and antibodies		
Saliva RT-PCR/PCR	Daily	Daily
Serology	Daily until D14; after D14, once or twice a week	Twice a week
Cerebral spinal fluid antibodies	Twice a week	Weekly
Spinal cerebral fluid	Cellularity and biochemistry twice a week	No comments
Tetrahydrobiopterin dosage (2mg/Kg/day)	Did not comment	15 after confirmation + another dosage 15 days after full dose replacement

*D1-D15: day 1, day 2, etc. **International Units/mL. ***Salt-losing brain syndrome.

Hyponatremia can also be a complication; the two most likely hypotheses are inappropriate antidiuretic hormone secretion syndrome (SSIHAD) or salt-losing brain syndrome (SCPS). When diagnosing SSIHAS, it is important to define normal fluid or hypervolemia, in which case, strict control of hydration becomes essential. It is important to collect uric acid to differentiate the diagnosis from SCPS; uric acid will be decreased in cases of SCPS. In SCPS, urinary sodium should be dosed, and the use of fludrocortisone or hydrocortisone should be considered.

The Milwaukee and Recife Protocols have many similarities in care related to rabies patients and are important for the treatment and care of patients in intensive care units. Both protocols indicate deep sedation, antiviral use, constant concern with electrolyte balance, and vasoconstriction related to the condition.

The Milwaukee Protocol highlights hyponatremia as a possible complication on the fifth day of hospitalization, and the use of prophylactic fludrocortisone may be indicated. The protocol points out that one of the causes of this disorder may be dehydration. This change may contribute to worsening brain injury, so recurrent sodium dosage is important. Both protocols recommend that serum sodium dosage should be performed twice a day.

Cerebral vasospasm is also associated with complications in patients with rabies. Both protocols recommend daily transcranial Doppler for the evaluation of this complication. In the Milwaukee Protocol, the recommendation is to perform trans-orbital ultrasonography for cases of intracranial hypertension. As a preventive measure against cerebral vasospasm, sapropterin or biopterin is used. Vasospasm in rabies cases is due to tetrahydrobiopterin (BH4) deficiency, which is essential for the synthesis of neural nitrous oxide (23), an important cerebral vasodilator. The Recife Protocol states that biopterin should be used as soon as the diagnosis of rabies is suspected. The Milwaukee Protocol states that sapropterin should be prescribed from the fifth day of hospitalization because this period is characterized by a higher risk of vasospasm and related complications. In the Brazilian protocol, BH4 dosage is recommended to evaluate biopterin activity at two times the fifteenth day of hospitalization and 15 days after replacement at maximum dose.

Another differing point in the protocols refers to sedation withdrawal. In the Milwaukee Protocol, sedation should be gradually removed after the eighth day, and on the twelfth day, the patient should be without sedation. In the Recife Protocol, in order to avoid immunomodulation, it is recommended to remove sedation according to the titers of neutralizing antibodies to the RABV in the cerebral spinal fluid. Specific rabies tests (serology and polymerase chain reaction [PCR]) are indicated repeatedly in both protocols. BH4 dosage is described in the Recife Protocol; this substance is associated with cerebral vasospasm in an angry patient. The Recife Protocol recommends the prophylactic use of biopterin, which must be maintained for four to six months, followed by a new dose of BH4 in the cerebrospinal fluid (CSF).

The rabies cases that were submitted to Milwaukee and Recife Protocols are shown in Table 2. Of the 39 total patients that, 11 survived; 6 patients had a dog bite, 1 was associated with a cat incident, while 4 occurred following exposure to bats. In only one case of the 39 surveyed here was the Recife Protocol used with success. The incubation period of the disease ranged from 22 days to 24 weeks, while in patients who did not survive this zoonosis, this time ranged from 23 days to 36 weeks.

**TABLE 2:** Survival of patients with human rabies treated with Milwaukee Protocol, from 2004 to 2019.

Sex	Age	Animal	T1	T2	Outcome	T3	Year	Country	M1	M2	M3	Induced coma	Virus	Reference
M	15	-	-	3	Death	26	2015	India	9	1	2	1	No information available	32
M	33	Dog	8	5	Death	8	2008	Canada	2	1	1	1	No information available	40
M	16	Bat	6	5	Death	7	2006	US (TX)	1	1	2	1	No information available	41
F	10	Bat	16	12	Death	15	2010	US (IND)	1	1	1	1	*Lasionycteris noctivagans,* bat variant	29
M	11	Dog	104	2	Death	27	2006	US (CA)	1	1	1	1	No information available	43
M	73	Bat	24	15	Death	64	2007	Canada	1	1	1	1	No information available	43
M	55	Dog	6	8**	Death	-	2009	Germany	1	1	2	1	No information available	44
F	34	Bat	3.5	6	Death	20	2007	Netherlands	1	1	1	1	*Duvenhage* bloodline	45
M	5	Dog	5	5	Death	22	2007	Eq. Guinea	1	1	1	1	Gabon canine rabies type virus	46
M	55	Bat	6	14***	Death	15	2009	US (MS)	1	1	2	1	No information available	47
F	9	Cat	4	6	Death	75	2015	Colombia	1	1	1	1	RABVs associated with vampire bats in Colombia	48,49
M	15	Bat	20	17	Death	16	2009	Colombia	1	1	1	1	RABV associated with the tricolor bat (*Perimyotis subflavus*)	50
F	37	Dog	96	10	Death	35	2010	UK	1	1	1	1	RABV variant found in Hematophagous bat	51
M	42	Dog	-	7	Death	24	2009	US (VA)	9	9	9	9	No information available	52
F	11	Cat	6	2	Death	-	2010	Romania	1	1	1	1	RABV found in Southeast Romania	31
F	41	Dog	11	6	Death	15	2012	Portugal	1	1	2	1	RABV lineage Africa 2 crane B	53
M	24	Dog	28	7	Death	17	2011	US (NY)	9	1	2	1	Canine RABV found in Afghanistan	54
M	63	Dog	12	7	Death	30	2011	US (MA)	9	1	2	1	RABV associated with bat motes (eared)	55
M	9	Monkey	5	18	Death	7	2011	BR (CE)	1	1	2	1	No information available	56
M	29	Dog	-	25	Death	35	2011	RSA	9	1	1	9	No information available	57
M	32	Dog	12	2	Death	14	2011	India	1	1	1	1	No information available	32
M	40	Dog	3.5	11	Death	22	2012	Italy	2	2	2	2	Arctic-like 1 lineage of the rabies virus (RABV) circulating in southern Asia, northern India, and the Middle East	58
M	41	Dog	-	-	Death	99	2012	Canada	9	9	9	9	RABV from the island of Hispaniola	59
F	58	Dog	36	4	Death	10	2014	UK	1	1	2	1	No information available	60
M	28	Dog	-	11	Death	22	2013	US (TX)	1	1	2	1	No information available	61
M	30	Dog	16	2	Death	36	2013	China	9	9	9	9	No information available	33
M	23	Dog	16	3	Death	11	2015	India	1	1	2	1	No information available	35
M	12	Dog	38	-	Death	13	2019	Saudi Arabia	9	9	9	1	No information available	62
F	15	Bat	4	5	S1	76	2004	US (WI)	1	1	1	1	No information available	12
M	15	Bat	4	7	S2	100	2008	Brazil (PE)	1	1	2	1	No information available	16
F	17	Bat	11	18	S1	34	2010	US (TX)	2	2	2	2	No information available	36
M	4	Dog	3	-	S3	50	2016	India	2	2	2	1	No information available	38
F	17	Dog	3	13	S1	90	2011	India	1	1	1	1	No information available	37,63
F	8	Cat	-	9	S1	36	2011	US (CA)	1	1	2	1	No information available	15
M	4	Dog	23	-	S3	90	2012	RSA	2	2	2	2	No information available	39
M	13	Dog	5	-	S3	-	2014	India	2	2	2	1	No information available	37
M	6	Dog	3	-	S3	-	2015	India	2	2	2	1	No information available	37
M	14	Bat	-	-	S1	40	2018	Brazil (AM)	1	1	1	1	No information available	42
M	8	Dog	20	8	S2	90	2019	India	9	9	9	9	No information available	64

1: used; 2: not used; 9: no information; T1 - incubation period; T2 - Time between onset of symptoms and initiation of treatment protocol (days); T3 - Time elapsed between hospitalization and death or hospital discharge (days); S1 - Survivor with few sequelae; S2 - Survivor with moderate sequelae; S3 - Survivor with severe sequelae; M1 - Amantadine; M2 - Ketamine; M3 - Ribavirin; **Vaccination performed one day before hospitalization. ***Protocol not initiated due to severity of neurological condition

## DISCUSSION

We compared the Milwaukee Protocol with the Recife Protocol for the treatment of rabies. A total of 39 cases of rabies, in which the anti-rabies treatment was used, were included in this study. Unfortunately, many cases of rabies, especially when patients die, are not published in the literature, even though the Milwaukee Protocol was used.

Vaccine and the use of immunoglobulin are contraindicated by both protocols. The development of antibodies against rabies in the central nervous system (CNS) is essential for curing the disease. Administration of immunoglobulin may delay this^12^. Sedation of patients with rabies should preferably be performed using midazolam and ketamine^17^. Ketamine is a noncompetitive antagonist of the N-methyl-D-aspartate (NMDA) receptor. This medication has an antiviral effect^18^.

Amantadine is a synthetic antiviral agent that can inhibit viral replication by binding to the NMDA antagonist receptor^19^. This drug is also indicated in both analyzed protocols. Other medications with antiviral activity, such as ribavirin and interferon, were indicated in the early versions of the Milwaukee Protocol. The withdrawal of these medications was due to the immunosuppressive effect they caused^20^.

A severe immune response in the central nervous system can cause damage and dysfunction, contributing to the death of patients. The cells present in the microglia present T-cell viral antigens that activate and produce cytokines and migration of mononuclear cells. An exaggerated inflammatory reaction may cause CNS damage. This damage causes the release of NO, which may be a neurotoxin^21^.

Ribavirin, a nucleotide analog, has the ability to inhibit RABV replication in vitro but has not shown benefit when used in cases of human rabies^22^. Amantadine and ketamine appear to play key roles in the treatment of rabies, and both medications have antiviral effects. Other drugs have also shown in vitro effects to inhibit RABV replication. Pyrimethamine has been shown to have antiviral effects in laboratory experiments, but its activity could not be replicated in animal models^23^. Favipiravir has been shown to be an antiviral agent with activity against various viruses such as arenavirus, bunyavirus, flavivirus, hand-foot-and-mouth disease, and influenza in in vitro and experimental animal models^24^. This drug has antiviral activity against RABV in vitro and in laboratory animal models^25^. There is a need for more data in preclinical research so that this medicine can be used in patients with human rabies.

Since 2004, the year in which the Milwaukee Protocol was first applied, the protocol has been used in 38 published cases (Table 2). The Recife Protocol was used only once. Among the described cases were 11 cases of patients who survived. Among these cases, the transmission of the disease occurred by dog incident in 6 cases, bat accident 4 times, and 1 case of transmission from a cat incident. Although the incubation period of rabies is a very controversial topic in the medical literature, in the presented cases it ranged from 4 weeks to more than 2 years. This incubation period may be related to the viral inoculum, virus concentration in the aggressor animal's saliva, bite location, and the centripetal migration capacity of the virus until it reaches the nerve roots. Bite location as well as injury severity are important factors in the evolution of the disease^6^ ^,^ ^7^.

Vaccination and immunoglobulin, contraindicated by the protocols, were used in some reports, most of them as prophylactic measures after exposure and prior to the onset of symptoms. Experimental studies with monkeys demonstrate that the administration of immunoglobulin, with or without rabies vaccine, induces a more rapid death, that is, an acceleration of the disease occurs in animals receiving immunoglobulin^26^. This early death may occur because the vaccine or immunoglobulin is of different strains than the wild virus to which the patient has been exposed. Rabies vaccines are usually made with several strains of virus, aimed at a robust immune response^27^. In patients with rabies disease, this inflammatory response can be harmful. The production of antibodies against rabies virus in peripheral blood does not mean that neutralizing antibodies, necessary for patients to evolve to a cure, will be produced in the CNS^28^. That is, the vaccine or immunoglobulin when administered to a patient with rabies virus disease, even though they are able to stimulate the production of antibodies, are not sufficient to alter the evolution of the disease in patients that already have the disease. Furthermore, the peripheral immunological response induced by the administration of vaccine or immunoglobulin has not been shown to improve the speed or quality of the appearance of antibodies in the CSF, and its effectiveness in these cases is questioned^12^ ^,^ ^13^. Someone who understands the pathophysiology of the disease might think that early antiviral treatment could result in better disease survival, but in the cases reported here, this fact was not true. Patients received treatment and medical support within only two days^29^ ^-^ ^33^ after the onset of symptoms, using ketamine and amantadine (medicines with antiviral action against RABV), and yet did not recover^34^.

In the case reports of patients who survived rabies, it is noted that 3 cases^35^ ^,^ ^36^ ^,^ ^37^ received rabies vaccination and immunoglobulin even after the onset of symptoms. The case presented as number 28 received an immunoglobulin dose one day after the onset of neurological symptoms of the disease. Perhaps immunoglobulin, when applied at the onset of symptoms, preferably during the period of centripetal migration of the virus to the dorsal nerve roots, may be important in treating the disease.

Patients who contract rabies, a disease with a high mortality rate and treatment protocols that are widely questioned in the medical literature, may require effective antiviral treatment that is administered early in the clinical course of the disease. The phase in which the virus reaches the CNS, causing encephalitis, may be too late for effective administration of available therapy. The best option for the treatment of rabies is still immunoglobulin-associated vaccination, both applied early in relation to the contact with an animal carrying the virus. When this therapeutic approach is not performed, the available drugs are inefficient in blocking the evolution of the disease^2^.

Among the patients who survived the disease and acquired it after an incident with infected animals, excluding cases of patients who acquired the virus after transplantation, none were older than 18 years; all were younger patients, aged 4 years to 17 years.

All patients who survived the rabies had a hospital stay longer than 30 days. This fact may be explained by the severity of the disease, requiring treatment in intensive care units and in-hospital rehabilitation care. Many patients still undergo a rehabilitation process at home or in specialized centers. There are reports of patients who remain bedridden and require home care due to disease sequelae^35^ ^,^ ^37^ ^-^ ^39^.

Patients still died of rabies due to protocol complications such as vasospasm, diabetes insipidus, and electrolyte disturbances, leading to arrhythmias and cardiorespiratory arrest. Many patients after death have high CSF-neutralizing antibody titers^29^, with no evidence of direct virus activity in the brain. Even patients who received treatment and started the protocol within two days after the onset of symptoms died^29^ ^-^ ^31^ ^,^ ^33^. Nine cases of surviving patients are presented, of which only one did not receive post-accident prophylaxis, considering vaccine or immunoglobulin as prophylaxis. This information contradicts the opinion of the two protocols cited before, that vaccine and immunoglobulin are not recommended because they can decrease the mortality of the disease.

The incubation time of rabies in the presented cases ranged from four weeks to more than two years. This incubation period may be related to the viral inoculum, virus concentration in the aggressor animal's saliva, and the centripetal migration capacity of the virus until it reaches the nerve roots. Bite location as well as injury severity are important factors in the evolution of the disease^6^ ^,^ ^7^.

In these 39 cases, the dog was still the main rabies-related mammal, followed by the bat (21 dog accidents and 9 bat accidents). This information confirms the importance of prophylaxis in treating animals carrying the virus, which may eventually transmit it to humans. The average incubation period for dog incidents was 27.4 weeks and for bat incidents was 10.5 weeks. This fact may be related to the greater severity of bat incidents, deeper wounds with greater viral inoculum, facilitating migration of the virus through peripheral nerves.

Most of the cases in which the main foundations of the Milwaukee Protocol were used occurred in developed countries. The country with the highest incidence of rabies in the world is India, with approximately 60,000 cases per year, but only 7 cases that occurred in India had publications on the use of the Milwaukee Protocol. A more accurate analysis of the effectiveness of this treatment needs to be evaluated in places where the incidence of rabies is high, such as India.

The Milwaukee Protocol has undergone several modifications from the first version to the current version (6.0), but the literature shows that further adjustment is needed^30^ ^,^ ^32^ ^,^ ^40^ ^,^ ^41^. In addition to supportive therapy in an intensive care unit, the use of effective antivirals seems to be a key point in treating rabies. Early treatment may have some influence on the outcome of the disease; the data presented does not confirm that the earlier the protocol is started, the greater the chances of survival.

The causes of death presented in the case reports justify the concern about vascular complications (vasospasm) and electrolyte disturbances; therefore, repetitive electrolyte collection and transcranial Doppler are of fundamental importance in the treatment of this disease.

The clinical presentation of rabies is very similar to that of other encephalitis; therefore, this disease may be more prevalent than it appears. Typical cases of the disease are unlikely to be confused with other conditions. The prolonged incubation period may make it more difficult for the physician to think about this diagnostic hypothesis. Many sites may have underdiagnosed cases of rabies, causing many patients to die without being properly diagnosed.

Some of the patients died after infectious conditions; in the articles, we did not find information on the use of antibiotic therapy. Most probably, several patients used this medication. Although neither of the protocols (Recife or Milwaukee) discuss in depth the risk of secondary bacterial infection, this seems to be a concern that should be present in all patients undergoing ICU therapy.

Of the patients who survived rabies, fewer than half were treated with amantadine, ketamine, or ribavirin. Of the surviving patients, 8 underwent induced coma as part of the rabies treatment. It is always worth remembering that treatment protocols cover a broad approach in patients, with care related to the most common complications and tests aimed at the early diagnosis of these complications and their correct treatment.

Among the surviving patients, all were hospitalized longer than 30 days, and many of them required outpatient follow-up until stabilization of neurological sequelae. This information makes the cost of treatment high, and the use of the protocol is somewhat limited in countries with worse socioeconomic conditions.

The last patient reported to have survived rabies occurred in Brazil in 2017. The patient had an accident with a bat, and two of his siblings, a boy and a girl, died of rabies. The patient came from a city located 399 km from the capital Manaus in the state of Amazonas, in the northern region of Brazil. The boy was admitted to the unit on December 2, 2018, and on December 9, showed signs of infection resolution^42^. The patient, admitted to the Manaus Institute of Tropical Medicine, underwent treatment based on the Milwaukee Protocol and survived with few sequelae. 

The protocols presented in this article indicate care in intensive treatment units and recommend several tests and the use of medications that are often inaccessible in places where rabies is highly prevalent. Evaluation of the effectiveness of the protocols is impaired, as is their acceptance in clinical practice. Many concepts can be reviewed to adapt protocols to different realities. We observed that the Milwaukee Protocol requires a large center for rabies treatment, but the disease occurs in places where resources are scarce. The Brazilian protocol, in trying to adapt the Milwaukee protocol for the reality of Brazil, did not succeed. This information can be demonstrated by analyzing the low adherence to the Recife Protocol, which would be more appropriate for treating rabies in places where the disease is most prevalent.

## References

[1] Deviatkin AA, Lukashev AN (2018). Recombination in the rabies virus and other lyssaviruses. Infect Genet Evol.

[2] Singh R, Singh KP, Cherian S, Saminathan M, Kapoor S, Manjunatha GB (2017). Rabies - epidemiology, pathogenesis, public health concerns and advances in diagnosis and control: a comprehensive review. Vet Q.

[3] Dietzschold B, Faber M, Schnell MJ (2003). New approaches to the prevention and eradication of rabies. Expert Rev Vaccines.

[4] Kuzmin IV, Hughes GJ, Botvinkin AD, Orciari LA, Rupprecht CE (2005). Phylogenetic relationships of Irkut and West Caucasian bat viruses within the Lyssavirus genus and suggested quantitative criteria based on the N gene sequence for lyssavirus genotype definition. Virus Res.

[5] Leung AKC, Davies HD, Hon KLE (2007). Rabies: epidemiology, pathogenesis, and prophylaxis. Adv Ther.

[6] Wilde H, Hemachudha T, Wacharapluesadee S, Lumlertdacha B, Tepsumethanon V (2013). Rabies in Asia: the classical zoonosis. Curr Top Microbiol Immunol.

[7] World Health Organization (WHO) (2013). WHO Expert Consultation on Rabies: Second Report.

[8] Wyatt J (2007). Rabies-update on a global disease. Pediatr Infect Dis J.

[9] Fooks AR, Banyard AC, Horton DL, Johnson N, McElhinney LM, Jackson AC (2014). Current status of rabies and prospects for elimination. Lancet.

[10] Shuangshoti S, Thorner PS, Teerapakpinyo C, Thepa N, Phukpattaranont P, Intarut N (2016). Intracellular Spread of Rabies Virus Is Reduced in the Paralytic Form of Canine Rabies Compared to the Furious Form. PLoS Negl Trop Dis.

[11] Jackson AC (2016). Human Rabies: a 2016 Update. Curr Infect Dis Rep.

[12] Willoughby RE, Tieves KS, Hoffman GM, Ghanayem NS, Amlie-Lefond CM, Schwabe MJ (2005). Survival after treatment of rabies with induction of coma. N Engl J Med.

[13] Ministério da Saúde (MS). Secretaria de Vigilância em Saúde (2009). Protocolo para tratamento de raiva humana no Brasil. Epidemiol & Serviços Saúde.

[14] World Health Organization (WHO) (2005). WHO Expert Consultation on Rabies: first report.

[15] Wiedeman J, Plant J, Glaser C, Messenger S, Wadford D, Sheriff H (2011). Recovery of a Patient from Clinical Rabies. MMWR.

[16] ProMED (2009). Rabies - Brazil (04): (PE), recovery.

[17] Lockhart BP, Tsiang H, Ceccaldi PE, Guillemer S (1991). Ketamine-mediated inhibition of rabies virus infection in vitro and in rat brain. Antivir Chem Chemother.

[18] Blanpied TA, Clarke RJ, Johnson JW (2005). Amantadine inhibits NMDA receptors by accelerating channel closure during channel block. J Neurosci.

[19] Anindita PD, Sasaki M, Okada K, Ito N, Sugiyama M, Saito-Tarashima N (2018). Ribavirin-related compounds exert in vitro inhibitory effects toward rabies virus. Antiviral Res.

[20] Consales CA, Bolzan VL (2007). Rabies review: immunopathology, clinical aspects and treatment. J Venom Anim Toxins Trop Dis.

[21] El-Sayed A (2018). Advances in rabies prophylaxis and treatment with emphasis on immunoresponse mechanisms. Int J Vet Sci Med.

[22] Appolinario CM, Jackson AC (2015). Antiviral therapy for human rabies. Antivir Ther.

[23] Rogée S, Larrous F, Jochmans D, Ben-Khalifa Y, Neyts J, Bourhy H (2019). Pyrimethamine inhibits rabies virus replication in vitro. Antiviral Res.

[24] Furuta Y, Komeno T, Nakamura T (2017). Favipiravir (T-705), a broad spectrum inhibitor of viral RNA polymerase. Proc Jpn Acad Ser B Phys Biol Sci.

[25] Banyard AC, Mansfield KL, Wu G, Selden D, Thorne L, Birch C (2019). Re-evaluating the effect of Favipiravir treatment on rabies virus infection. Vaccine.

[26] Willoughby RE (2009). "Early death" and the contraindication of vaccine during treatment of rabies. Vaccine.

[27] Faber M, Li J, Kean RB, Hooper DC, Alugupalli KR, Dietzschold B (2009). Effective preexposure and postexposure prophylaxis of rabies with a highly attenuated recombinant rabies virus. Proc Natl Acad Sci U S A.

[28] Dietzschold B, Kao M, Zheng YM (1992). Delineation of putative mechanisms involved in antibody-mediated clearance of rabies virus from the central nervous system. Proc Natl Acad Sci U S A.

[29] Christianson JA, Davis BM, Kruger L, Light AR (2010). The Role of Visceral Afferents in Disease. Translational Pain Research: From Mouse to Man.

[30] Aramburo A, Willoughby RE, Bollen AW, Glaser CA, Hsieh CJ, Davis SL (2011). Failure of the Milwaukee protocol in a child with rabies. Clin Infect Dis.

[31] Luminos M, Barboi G, Draganescu A, Cercel A, Staniceanu F, Jugulete G (2011). Human Rabies in a Romanian girl - an ante mortem case study. Rabies Bull Eur.

[32] Manesh A, Mani RS, Pichamuthu K, Jagannati M, Mathew V, Karthik R (2018). Case Report: Failure of Therapeutic Coma in Rabies Encephalitis. Am J Trop Med Hyg.

[33] Chen Y-G, Kan L-P, Lee C-H, Lin S-H, Chu D-M, Chang F-Y (2015). Symptomatic hypercalcemia in a rabies survivor underwent hemodialysis. Hemodial Int.

[34] Hemachudha T, Ugolini G, Wacharapluesadee S, Sungkarat W, Shuangshoti S, Laothamatas J (2013). Human rabies: neuropathogenesis, diagnosis, and management. Lancet Neurol.

[35] Karande S, Muranjan M, Mani RS, Anand AM, Amoghimath R, Sankhe S (2015). Atypical rabies encephalitis in a six-year-old boy: clinical, radiological, and laboratory findings. Int J Infect Dis.

[36] Holzmann-Pazgal G, Wanger A, Degaffe G, Rose C, Heresi G, Amaya R (2010). Presumptive Abortive Human Rabies --- Texas, 2009. MMWR.

[37] de Souza A, Madhusudana SN (2014). Survival from rabies encephalitis. J Neurol Sci.

[38] Manoj S, Mukherjee A, Johri S, Kumar KVSH (2016). Recovery from rabies, a universally fatal disease. Mil Med Res.

[39] Weyer J, Msimang-Dermaux V, Paweska JT, Roux K le, Govender P, Coertse J (2016). A case of human survival of rabies, South Africa. South Afr J Infect Dis.

[40] McDermid RC, Saxinger L, Lee B, Johnstone J, Gibney RTN, Johnson M (2008). Human rabies encephalitis following bat exposure: failure of therapeutic coma. Can Med Assoc J.

[41] ProMED (2006). Rabies, human - USA (TX).

[42] SUSAM. Secretaria de Estado da Saúde do Amazonas (2018). Amazonas registra o segundo caso de sobrevivência por raiva humana no Brasil.

[43] Johnstone J, Saxinger L, McDermid R, Bagshaw S, Resch L, Lee B (2008). Human Rabies --- Alberta, Canada. 2007. MMWR.

[44] Schmiedel S, Panning M, Lohse A, Kreymann KG, Gerloff C, Burchard G (2007). Case report on fatal human rabies infection in Hamburg, Germany, March 2007. Euro Surveill.

[45] van Thiel PPAM, de Bie RMA, Eftimov F, Tepaske R, Zaaijer HL, van Doornum GJJ (2009). Fatal Human Rabies due to Duvenhage Virus from a Bat in Kenya: Failure of Treatment with Coma-Induction, Ketamine, and Antiviral Drugs. PLoS Negl Trop Dis.

[46] Rubin J, David D, Willoughby RE, Rupprecht CE, Garcia C, Guarda DC (2009). Applying the Milwaukee protocol to treat canine rabies in Equatorial Guinea. Scand J Infect Dis.

[47] Turabelidze G, Pue H, Grim A, Patrick S (2009). First human rabies case in Missouri in 50 years causes death in outdoorsman. Mo Med.

[48] Caicedo Y, Paez A, Kuzmin I, Niezgoda M, Orciari LA, Yager PA (2015). Virology, immunology and pathology of human rabies during treatment. Pediatr Infect Dis J.

[49] Jackson AC, Garland A (2015). Fatal Rabies Case Did not Die “Accidentally” and Should not Be Considered a Rabies Survivor. Pediatr Infect Dis J.

[50] Badillo R, Mantilla JC, Pradilla G (2009). [Human rabies encephalitis by a vampire bat bite in an urban area of Colombia]. Biomed Rev Inst Nac Salud.

[51] Hunter M, Johnson N, Hedderwick S, McCaughey C, Lowry K, McConville J (2010). Immunovirological correlates in human rabies treated with therapeutic coma. J Med Virol.

[52] Troell P, Miller-Zuber B, Ondrush J, Murphy J, Fatteh N, Feldman K (2010). Human Rabies --- Virginia, 2009. MMWR.

[53] Santos A, Cale E, Dacheux L, Bourhy H, Gouveia J, Vasconcelos P (2012). Fatal case of imported human rabies in Amadora, Portugal, August 2011. Euro Surveill.

[54] Javaid W, Amzuta IG, Nat A, Johnson T, Grant D, Rudd RJ (2012). Imported Human Rabies in a U.S. Army Soldier - New York, 2011. MMWR.

[55] Greer DM, Robbins GK, Lijewski V, Gonzalez RG, Gonzales RG, McGuone D (2013). Case records of the Massachusetts General Hospital. Case 1-2013. A 63-year-old man with paresthesias and difficulty swallowing. N Engl J Med.

[56] ProMED (2012). Rabies, human - Brazil (03): (CE).

[57] ProMED (2012). Rabies - South Africa (03): (NL).

[58] De Benedictis P, Perboni G, Gentili C, Gaetti L, Zaffanella F, Mutinelli F (2012). Fatal case of human rabies imported to Italy from India highlights the importance of adequate post-exposure prophylaxis, October 2011. Euro Surveill.

[59] Branswell H, Globe and Mail (2012). Testing suggests Toronto rabies case infected in Dominican Republic.

[60] Pathak S, Horton DL, Lucas S, Brown D, Quaderi S, Polhill S (2014). Diagnosis, management and post-mortem findings of a human case of rabies imported into the United Kingdom from India: a case report. Virol J.

[61] Wallace RM, Bhavnani D, Russell J, Zaki S, Muehlenbachs A, Hayden-Pinneri K (2014). Rabies Death Attributed to Exposure in Central America with Symptom Onset in a U.S. Detention Facility - Texas, 2013. MMWR.

[62] Alknawy M, Mohammed I, Ulla SN, Aboud AA (2018). First confirmed case of human rabies in Saudi Arabia. IDCases.

[63] Netravathi M, Udani V, Mani R, Gadad V, Ashwini M, Bhat M (2015). Unique clinical and imaging findings in a first ever documented PCR positive rabies survival patient: A case report. J Clin Virol.

[64] Bokade CM, Gajimwar VS, Meshram RM, Wathore SB (2019). Survival of Atypical Rabies Encephalitis. Ann Indian Acad Neurol.

